# *In silico* prediction of the granzyme B degradome

**DOI:** 10.1186/1471-2164-12-S3-S11

**Published:** 2011-11-30

**Authors:** Lawrence JK Wee, Esmond PS Er, Lisa FP Ng, Tong

**Affiliations:** 1 Data Mining Department, Institute for Infocomm Research, 1 Fusionopolis Way, #21-01 Connexis South Tower, Singapore 138632; 2 Singapore Immunology Network, 8A Biomedical Grove, #04-06 Immunos, Biopolis, Singapore 138648; 3 Department of Biochemistry, Yong Loo Lin School of Medicine, National University of Singapore, 8 Medical Drive, Singapore 117597

## Abstract

**Background:**

Granzyme B is a serine protease which cleaves at unique tetrapeptide sequences. It is involved in several signaling cross-talks with caspases and functions as a pivotal mediator in a broad range of cellular processes such as apoptosis and inflammation. The granzyme B degradome constitutes proteins from a myriad of functional classes with many more expected to be discovered. However, the experimental discovery and validation of bona fide granzyme B substrates require time consuming and laborious efforts. As such, computational methods for the prediction of substrates would be immensely helpful.

**Results:**

We have compiled a dataset of 580 experimentally verified granzyme B cleavage sites and found distinctive patterns of residue conservation and position-specific residue propensities which could be useful for *in silico* prediction using machine learning algorithms. We trained a series of support vector machines (SVM) classifiers employing Bayes Feature Extraction to predict cleavage sites using sequence windows of diverse lengths and compositions. The SVM classifiers achieved accuracy and A_ROC_ scores between 71.00% to 86.50% and 0.78 to 0.94 respectively on independent test sets. We have applied our prediction method on the Chikungunya viral proteome and identified several regulatory domains of viral proteins to be potential sites of granzyme B cleavage, suggesting direct antiviral activity of granzyme B during host-viral innate immune responses.

**Conclusions:**

We have compiled a comprehensive dataset of granzyme B cleavage sites and developed an accurate SVM-based prediction method utilizing Bayes Feature Extraction to identify novel substrates of granzyme B *in silico*. The prediction server is available online, together with reference datasets and supplementary materials.

## Background

Proteolysis - the specific and limited cleavage of proteins by enzymes called proteases - represents an important mechanism for post-translational control in all living organisms [1]. Granzymes (short for granule enzymes) belong to a unique class of serine proteases which are known to mediate critical roles in the innate immune response against virus-infected or tumor cells through the induction of apoptotic cell death [2]. Consequently, the enzymes have been implicated in the pathogenesis of several chronic inflammatory and cardiovascular disorders. Granzymes are released into the cytoplasm of the target cells through endocytosis of cytolytic granules released by cytotoxic T cells or natural killer cells [2]. Once released into the target cells, granzymes go on to cleave specific cellular proteins and activate multiple signaling pathways leading to apoptotic cell death. Of the five human subtypes discovered to date (granzymes A, B, H, K and M), granzyme B has been the most well studied. Like caspases, granzyme B recognizes specific tetrapeptide sequence motifs (P_4_-P_3_-P_2_-P_1_) and cleave proteins after aspartate residue at P_1 _[3,4]. Besides cleaving specific proteins regulating apoptotic cell death, granzyme B has been reported to cleave proteins across a wide spectrum of other functional classes, ranging from nuclear and cytoskeletal components to membrane receptors and viral proteins [5].

To date, more than 500 granzyme B substrates have been characterized and many more are expected to be identified [5]. While systematic experimental discovery and validation of bona fide substrates are necessary for elucidating the granzyme B degradome, many of the processes are often time consuming and laborious. For these reasons, computational prediction of substrates could be immensely helpful in generating initial hypotheses and experimental leads. While a wide range of computational methods have been applied for substrate prediction of related proteases such as caspases [6,7], only a limited number are available for prediction of granzyme B substrates. PeptideCutter [8] is a general protease substrates cleavage prediction server which predicts for potential granyzme B cleavage sites using preferential tetrapeptide cleavage (P_4_-P_3_-P_2_-P_1_) specificities derived from *in vitro* combinatorial library studies by Thornberry *et al*. [4]. Backes *et al*. developed the GraBCas software which extended the use of the *in vitro* specificities by incorporating position-specific scoring matrices and accounting for conserved residues at P_1_' and P_2_' positions [9]. More recently, Barkan *et al*. advanced the field through the application of the support vector machines (SVM) method on a set of experimentally verified cleavage sites using both sequence and structural features [10].

In this paper, we have compiled a dataset of 580 experimentally verified granzyme B cleavage sites and found distinctive patterns of residue conservation and position-specific residue propensities which could be useful for *in silico* prediction using machine learning algorithms. We trained a series of SVM classifiers employing Bayes Feature Extraction to predict cleavage sites using sequence windows of diverse lengths and compositions. The SVM classifiers achieved accuracy and A_ROC_ scores between 71.00% to 86.50% and 0.78 to 0.94 respectively on independent test sets. We applied our prediction method on the Chikungunya viral proteome and identified several regulatory domains of viral proteins to be potential sites of granzyme B cleavage, suggesting direct antiviral activity of granzyme B during host-viral innate immune responses. A web server, together with reference datasets and supplementary materials, can be accessed at http://www.casbase.org/grasvm/index.html.

## Results and discussion

### Sequence analysis of granzyme B cleavage sites

Using peptide combinatorial libraries, Thornberry and co-workers had previously identified the presence of distinctive sequence specificities governing protein cleavage of both caspase and granzyme B substrates [4]. In particular, specific tetrapeptide sequences upstream of the cleavage site (P_4_-P_3_-P_2_-P_1_) of granzyme B targets serve as recognition sites for protein cleavage. The tetrapeptide “IEPD” was identified as the optimal tetrapetide cleavage sequence *in vitro*. However, emerging data on granzyme B substrates suggest that the *in vivo* cleavage specificities are far more diverse, with numerous substrates possessing cleavage specificities extending beyond the tetrapeptide sequence [5,10].

We compiled a comprehensive dataset of 580 unique granzyme B cleavage sites extracted from experimentally verified substrates as reported in literature. Data was extracted from the substrates list compiled in Barkan *et al*. [10], as well as the proteomic studies by Van Damme *et al*. [5]. In addition to the P_4_P_1_ cleavage site sequences, segments of different lengths and compositions centered on the P_1_ position were selected. In all, eight groups of sequences were obtained - P_2_P_2_^’^, P_4_P_1_, P_4_P_2_^’^, P_4_P_4_^’^, P_6_P_6_’, P_8_P_8_^’^, P_10_P_10_’ and P_14_P_10_'. We further extracted an equal number of “non-cleavage” sites by randomly selecting non-annotated tetrapeptide sequences (and other corresponding sequence segments) on the substrates. On the P_10_P_10_’ dataset, we computed P_x_ (or relative position-specific residue propensity) of each amino acid at the different residue positions along the 20-mer sequence. P_x_ was computed as the ratio of the frequency of occurrence of a particular residue in the cleavage site sequences over the same residue in the non-cleavage site sequences at the particular position.

**Figure 1:**
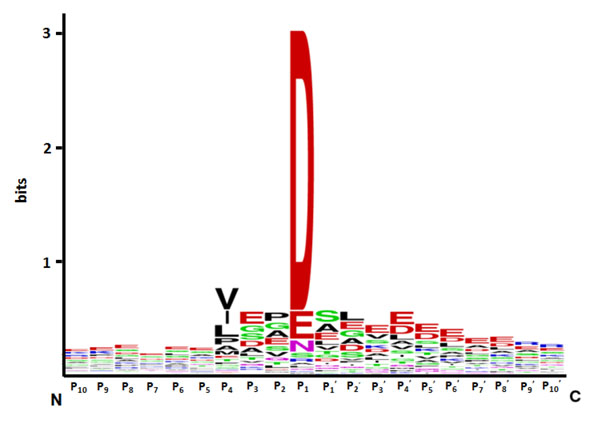
Sequence logo of amino acids in the vicinity of the granzyme B cleavage site (P_10_ to P_10_^’^)

**Figure 2:**
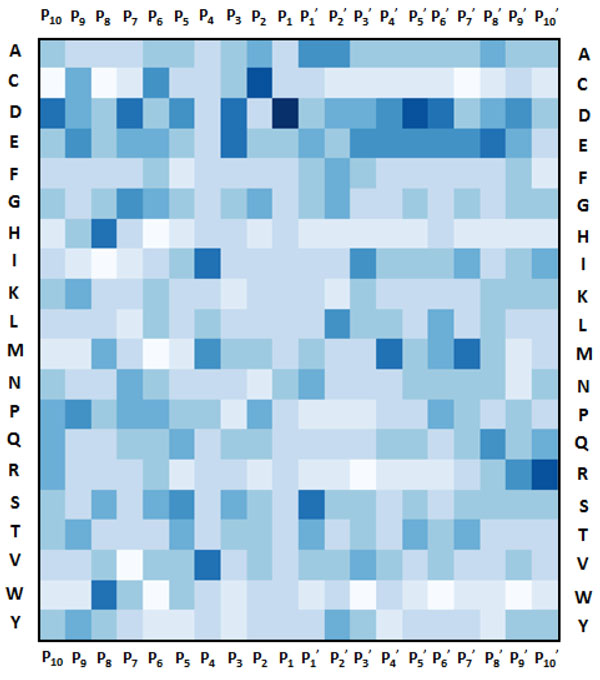
**Heat map of relative position-specific amino acid propensities (P_x_)**. P_x_ values were computed for P_10_P_10_^’^ dataset. P_x_ values were computed as the ratio of the frequency of occurrence of the amino acid in the cleavage sites pool over the frequency of occurrence of the same amino acid in the non-cleavage sites pool at a specific position. Increasing color intensities (white to blue) indicate proportionately greater enrichment of the amino acid in the cleavage sites over non-cleavage sites, and vice versa for decreasing color intensities.

As shown in Table 1, measurements of average P_x_ in the P_10_P_10_’ sequences indicate an unusually high enrichment for the negatively charged amino acids Asp and Glu with average P_x_ scores of 1.98 and 1.46 respectively. Conversely, there are significantly lower propensities for the positively charged amino acids (His, Lys and Arg all possess average P_x_ of less than 0.70). In addition, the large hydrophobic residue Trp is also weakly represented among the cleavage site sequences, with average P_x_ of 0.46. To further quantify position-specific residue propensities, we plotted a sequence logo using the P_10_P_10_’ sequences and constructed a heatmap of P_x_ scores from the same dataset (as shown in Figures 1 and 2 respectively). At P_1_ position, Asp is expectedly the most conserved residue, with notable presence of Glu, Asn and Ser as alternatives. Interestingly, Pro and Cys residues are more conserved in the cleavage sites compared to the non-cleavage sites at P_2_ position, while P_3_ is dominated by the acidic residues Asp and Glu. The P_4_ position showed significant propensities for the branched-chain amino acids Leu, Ile and Val. Remarkably, the most prominent feature distinguishing cleavage site sequences from non-cleavage site sequences appear to be the extended stretches of acidic residues (Asp and Glu) upstream and downstream of the cleavage site. Downstream of the cleavage site, it is further observed that small amino acids such as Gly, Ser, Ala and Leu are highly enriched at P_1_^’^ and P_2_^’^. These results indicate that cleavage sites of granzyme B substrates and the flanking upstream and downstream sequences have unique position-specific residue propensities. These composite signatures could be incorporated into machine learning algorithms for the development of accurate computational prediction models.

**Table 1:** Average P_x_ of amino acids: Average P_x_ of each amino acid was calculated by averaging the P_x_ values of the particular amino acid across all residue positions within the 20-mer sequence window (P_10_P_10_^’^)

Amino acid	Average P_x_
A	1.14
C	0.72
D	1.98
E	1.46
F	0.80
G	1.02
H	0.48
I	1.05
K	0.69
L	0.88
M	1.10
N	0.86
P	0.93
Q	0.96
R	0.66
S	1.07
T	0.96
V	1.08
W	0.46
Y	0.80

### SVM prediction of granzyme B cleavage sites

To account for these unique signatures of residue conservation and position-specific propensities for *in silico* prediction, we developed SVM prediction models incorporating the Bayes Feature Extraction (BFE) approach as described in Shao *et al.*[*11*]. Vector representation using the BFE approach was shown to significantly improve performance in several bio-computational problems - such as the prediction of protein methylation sites [11], caspase cleavage [12] and linear B-cell epitopes [13] - over simple binary encoding schemes. In BFE, feature vectors encoded in a bi-profile manner comprising of positive position-specific and negative position-specific profiles. These profiles were generated by accounting for the frequency of occurrence of each amino acid at each position of the sequences in the positives pool (cleavage site sequences) and negatives pool (non-cleavage site sequences) respectively. Here, we trained a series of SVM classifiers on sequence windows of diverse lengths and compositions (P_2_P_2_’, P_4_P_1_, P_4_P_2_ , P_4_P_4_’, P_6_P_6_’, P_8_P_8_’, P_10_P_10_’ and P_14_P_10_') using simple binary encoding and BFE schemes (details in Materials and Methods). Datasets were segmented into training and independent test sets comprising of 480 positives/480 negatives and 100 positives/100 negatives respectively. Using the RBF kernel, 10-fold cross-validation was implemented to acquire the optimal set of C and γ parameter values. SVM classifiers were subsequently trained on the entire training set using the optimized parameters and evaluated on the independent test sets.

As given in Table 2, the P_4_P_1_ classifier utilizing simple binary encoding (P_4_P_1_-SVM) registered an accuracy of 77.50% and A_ROC_ of 0.77 on independent testing. The other classifiers observed consistent improvement in accuracy and A_ROC_ as the sequence window extends beyond P_4_P_1_ to include the flanking upstream and downstream residues, achieving the best scores of 83.50% and 0.89 respectively with the P_8_P_8_^’^-SVM classifier. The P_4_P_1_ classifier utilizing BFE scheme (P_4_P_1_-Bayes) attained an accuracy of 76.50% and A_ROC_ of 0.84 (Table 3). In a similar fashion, prediction performance improved steadily as the sequence window is extended beyond P_4_P_1,_ achieving the best accuracy of 86.50% with the P_8_P_8_^’^-Bayes classifier and the best A_ROC_ of 0.94 with the P_10_P_10_^’^-Bayes and P_14_P_10_^’^-Bayes classifiers. Interestingly, in both feature representation schemes, prediction performances did not significantly improve with sequences longer than P_8_P_8_^’^. This could be due to that fact that much of the information specific for differentiating cleavage sites from non-cleavage sites are encoded within the sequences situated closer to the cleavage sites, as evidenced by the unique residue propensities discussed earlier. In addition, accuracy and A_ROC_ scores across most sequence lengths and compositions were generally higher for classifiers trained using the BFE scheme, with the greatest improvements observed when longer sequences (P_6_P_6_^’^, P_8_P_8_^’^, P_10_P_10_^’^ and P_14_P_10_^’^) were employed.

**Table 2:** Results of SVM prediction using simple binary encoding

SVM classifier	Sensitivity (%)	Specificity (%)	Accuracy (%)	A_ROC_
P_2_P_2_^’^-SVM	73.00	68.00	70.50	0.77
P_4_P_1_-SVM	77.00	78.00	77.50	0.85
P_4_P_2_^’^-SVM	85.00	76.00	80.50	0.89
P_4_P_4_^’^-SVM	84.00	80.00	82.00	0.89
P_6_P_6_^’^-SVM	84.00	82.00	83.00	0.89
P_8_P_8_^’^-SVM	83.00	84.00	83.50	0.89
P_10_P_10_^’^-SVM	81.00	82.00	81.50	0.89
P_14_P_10_^’^-SVM	78.00	81.00	79.50	0.88

**Table 3:** Results of SVM prediction using Bayes Feature Extraction

SVM classifier	Sensitivity (%)	Specificity (%)	Accuracy (%)	A_ROC_
P_2_P_2_^’^-Bayes	71.00	71.00	71.00	0.78
P_4_P_1_-Bayes	79.00	74.00	76.50	0.84
P_4_P_2_^’^-Bayes	82.00	80.00	81.00	0.89
P4P4’-Bayes	82.00	81.00	81.50	0.91
P_6_P_6_^’^-Bayes	86.00	84.00	85.00	0.91
P_8_P_8_^’^-Bayes	89.00	84.00	86.50	0.93
P_10_P_10_^’^-Bayes	87.00	85.00	86.00	0.94
P_14_P_10_^’^-Bayes	88.00	82.00	85.00	0.94

Next, we compared our prediction method with GraBCas [9] and the SVM models developed by Barkan *et al*. [10]. As the GraBCas algorithm primarily focuses on the detection of specific tetrapeptide motifs, we applied the algorithm on our P_4_P_1_ independent test set which contains only the tetrapeptide cleavage site sequences. Using the recommended cut-off score of 0.12, GraBCas predicted only 61 out of 100 cleavage sites correctly (*S_n_*=61%). On the same dataset, our P_4_P_1_-SVM and P_4_P_1_-Bayes classifiers respectively predicted 77 out of 100 (*S_n_*=77%) and 79 out of 100 (*S_n_*=79%) cleavage sites correctly. The weaker sensitivity scores observed for GraBCas could be due to the utilization of position-specific scoring matrices (PSSMs) which are derived from a small, out-dated set of *in vitro* cleavage specificities and the absolute requirement of Asp residue at P_1_ on the cleavage sites. To further evaluate the performance of the PSSM-based algorithm in our context, we constructed PSSMs derived from our entire dataset of cleavage sites, and found that the A_ROC_ scores of the PSSM-based predictors were generally poorer than our SVM-based classifiers (data not shown). In Barkan *et al*., the best SVM classifier recorded a true positive rate (TPR) of 0.79 and false positive rate (FPR) of 0.21 at the critical point on the receiver operating characteristic (ROC) curve when tested on an independent test set. In our SVM method, several classifiers encoded using the BFE scheme registered better prediction performance when measured by the same metrics; P_10_P_10_^’^-Bayes with TPR of 0.86 and FPR of 0.14, as well as P_14_P_10_^’^-Bayes, P_8_P_8_^’^-Bayes and P_6_P_6_^’^-Bayes with TPRs of 0.85 and FPRs of 0.15.

### Prediction of granzyme B cleavage of CHIKV proteome

To investigate the applicability of our computational method, we applied the SVM classifiers on the proteome of the Chikungunya virus (CHIKV) and analyzed for the presence of hitherto undiscovered granzyme B cleavage sites. CHIKV is a member of the alphavirus family and has been known to be transmitted to humans via the bite of the virus-borne Aedes mosquito [14]. Acute infection of CHIKV results in symptoms such as abrupt fever, skin rash and arthralgia. As CHIKV epidemics have been re-emerging in recent times, there have been concerted efforts directed toward developing relevant vaccines and drug therapies. During viral infections, granzyme B has been reported to mediate downstream cleavage of critical host regulatory proteins, leading to the induction of the apoptotic cell death, and hence disruption of viral propagation [15]. Although granzyme B-induced apoptotic cell death has long been considered the *de facto* mechanism for killing virus-infected cells, emerging evidence suggest that the enzyme could exert direct antiviral activity through cleavage of the viral proteins [15]. For these reasons, it is intuitive to speculate if the CHIKV proteome may be directly regulated by granzyme B activity in this manner and if cleavage of specific CHIKV proteins will potentiate the host innate immune responses against viral infectivity.

Four non-structural and four structural proteins of the CHIKV proteome (strain: LR2006_OPY1) were predicted for granzyme B cleavage sites using the P_8_P_8_’-Bayes classifier. Since the majority of experimentally verified cleavage sites were known to be cleaved after the Asp residue, we have restricted our prediction scans to only cleavage sites containing Asp residue at P_1_. As shown in Table 4, we found potential granzyme B cleavage sites in all CHIKV proteins except the structural proteins E1, E3 and 6K. A significantly larger proportion of these sites were found in the non-structural proteins NSP1, NSP2, NSP3 and NSP4, as compared to the structural proteins E2 and capsid. As the alphaviral non-structural proteins are known to be involved in viral survival and replication, we would expect the cleavage of these proteins by granzyme B to abrogate viral survival mechanisms at different points of the viral reproduction cycle [16]. Indeed, the cleavage of NSP1 protein at Asp-11 and Asp-58, which are both localized within the methyltransferase domain, could lead to inhibition of the mRNA capping during RNA synthesis. Conversely, the cleavage of NSP2 helicase domain at Asp-247 and Asp-343, as well as the RNA polymerase domain at Asp-291, Asp-371, Asp-476 and Asp-540 on the NSP4 protein could hinder viral RNA synthesis and translation. In addition, cleavage of the capsid protein at Asp-112 and Asp-114 within the protease domain might lead to prevention of auto-cleavage of the immature capsid protein from the viral structural polyprotein.

**Table 4:** Prediction of granzyme B cleavage of CHIKV proteome

Protein	Biological activity and function	Cleavage sites*
NSP1	Non-structural: mRNA capping	9, 11, 58, 525
NSP2	Non-structural: NTPase, helicase and protease activities	116, 247, 343
NSP3	Non-structural: ADP-ribose phosphatase activity	181, 350, 363, 506
NSP4	Non-structural: RNA polymerase activity	219, 371, 476, 540
E1	Structural: virus-host cell fusion	Nil
E2	Structural: virus-host cell attachment	77
E3	Structural: unknown	Nil
Capsid	Structural: protease, viral nucleocapsid formation	112, 174
6K	Structural: membrane permeabilization, budding of viral particles	Nil

*Position of the P_1_ residue on the substrate. All predicted cleavage sites contain Asp at P_1_. *Underlines* indicate P_1_ location in the functional domain(s) of protein.

## Conclusions

In this paper, we constructed a comprehensive database of experimentally verified granzyme B cleavage sites for analysis and development of prediction methods. We discovered that flanking sequences of cleavage sites possess distinctive residue composition and position-specific propensity patterns which could be helpful in discriminating the cleavage sites from non-cleavage sites in *silico*. We have rigorously tested SVM classifiers employing simple binary encoding and the Bayes Feature Extraction schemes to predict granzyme B cleavage sites. Results also show that the best classifiers are more effective than existing algorithms. We applied our prediction method on the Chikungunya viral proteome and identified several regulatory domains of viral proteins to be potential targets of granzyme B cleavage, suggesting a direct antiviral function of granzyme B during host-viral innate immune responses. To complement experimental research, we have implemented our prediction method on a web server which is freely accessible at http://www.casbase.org/grasvm/index.html. In the immediate future, we will be exploring the influence of cleavage site secondary structures, solvent accessibilities and other physicochemical properties on protease-substrate cleavage specificities, as well as their potential for enhancing the performance of our SVM prediction models. Computational prediction of granzyme B substrates will complement on-going experimental efforts and refine our understanding of the biochemistry of this fascinating protease and its relatives.

## Materials and methods

### Datasets

We extracted a pool of 779 unique, experimentally verified cleavage sites from literature. 723 sequences were derived from proteomic experimental studies conducted by Van Damme *et al*. [5], with the remaining 56 from systematic *in vitro* and *in vivo* experiments as compiled in Barkan *et al*. [10]. We further extracted sequence segments of different lengths flanking the P_1_ cleavage sites. In all, eight datasets were constructed: the tetrapeptide cleavage site sequences (referred to as P_4_P_1_ dataset) and sequences containing residues extended to P_14_ and P_10_' (P_2_P_2_^’^, P_4_P_2_^’^, P_4_P_4_^’^, P_6_P_6_’, P_8_P_8_^’^, P_10_P_10_’ and P_14_P_10_' datasets). These sequences were assigned as positive examples for analysis as well as for development of the SVM method. An equal number of “non-cleavage sites” or negative examples were obtained by randomly extracting P_1_ residues on the substrates. Sequence segments of the aforementioned lengths and compositions were obtained as detailed earlier. All datasets of positive and negative sequences (779/779) were subsequently subjected to homology filtering using the CD-HIT clustering algorithm [17] where sequences bearing more than 85% sequence identity with any other sequence in the dataset were eliminated. The final datasets comprised of 580 positive and 580 negative sequences (the complete list of cleavage sites is available in Additional File 1). For analysis, all 580 positives and 580 negatives from the P_10_P_10_’ dataset were used. For SVM model development, datasets were partitioned into training and test sets consisting of 480 positives/480 negatives and 100 positives/100 negatives respectively.

### Sequence analysis

The relative position-specific residue propensity P_x_ was computed as the ratio of the frequency of occurrence of a particular amino acid in the cleavage sites pool to its frequency of occurrence in the non-cleavage sites pool at a specific position on the sequence. Using the P_10_P_10_’ dataset, P_x_ scores were calculated for every amino acid at each of the twenty residue positions and visualized on heat maps. Additionally, we constructed a sequence logo representation of the positive sequences from the P_10_P_10_^’^ dataset using WebLogo [18].

### SVM vector representation

To encapsulate sequence information for SVM training and testing, input vectors were constructed using simple binary or bi-profile Bayes Features encoding. For simple binary encoding, each amino acid is represented by a vector of 20 dimensions, comprising of binary values of zeroes and ones. For example, alanine was represented as [0,0,0,0,0,0,0,0,0,0,0,0,0,0,0,0,0,0,0,1] and cysteine as [0,0,0,0,0,0,0,0,0,0,0,0,0,0,0,0,0,0,1,0]. Hence, in this case, a 20-mer sequence will be represented by a vector of 400 dimensions (20 x 20). Detailed description on bi-profile vector encoding using Bayes Features is available in Shao *et al*. [11]. In short, feature vectors contain information from both positive position-specific and negative position-specific profiles. These profiles were generated by accounting for the frequency of occurrence of each amino acid at each position of the sequences in the positives pool (cleavage site sequences) and negatives pool (non-cleavage site sequences) respectively. Therefore, a 20-mer sequence (from the P_10_P_10_’ dataset) would be represented by a feature vector of 40 dimensions (20 x 2), containing information of the residues in both positive (cleavage site sequences) and negative (non-cleavage site sequences) spaces. For all sequence representations, P_1_ residues were excluded from the feature vectors.

### SVM model development

To train and test the SVM models, we used the LIBSVM package provided by Chang and Lin [19]. For details on the SVM method, readers are advised to consult the article by Burges [20]. In short, SVM is grounded on the structural risk minimization concept from statistical learning theory. A set of training examples (positives and negatives) can be encoded by the feature vectors *x*_i_ (*i* = 1, 2,….N ) with resultant classes *y_i_* ∈ {+1,-1}. The SVM algorithm trains a classifier by representing the input feature vectors, using a kernel function in the majority of cases, onto a high-dimensional space, and then selects a discriminating hyperplane that separates the two classes with maximal margin and the least error. The decision function for classification of unseen examples is defined as:

where K (*x_i_*·*x_j_* ) is the kernel function, and the parameters are resolved by maximizing the following:

with the following constraints:

*C* is the regularization variable that directs the trade-off between margin and classification error. We used the radial basis function (RBF) kernel and performed grid-based optimization for γ, which controls the capacity of the RBF kernel, and *C* using 10-fold cross-validation. In 10-fold cross-validation, the training set was randomly partitioned into ten subsets where one of the subsets was used as the test set while the other subsets were used for training the classifier. The trained classifier was evaluated using the test set. This procedure was repeated ten times using different subsets for testing, hence making sure that all subsets were utilized for both training and testing. The optimized γ and C values were applied towards training the entire training set to generate the SVM classifier for independent testing on an out-of-sample test set. Graphical plots of optimization results are provided in Additional File 2.

### Evaluation of model performance

A set of statistical variables were established to evaluate the performance of the SVM classifier for the prediction of granzyme B cleavage sites:

(i) True Positives (*TP*), for the number of correctly classified cleavage sites.

(ii) False Positives (*FP*), for the number of incorrectly classified non-cleavage sites.

(iii) True Negatives (*TN*), for the number of correctly classified non-cleavage sites.

(iv) False Negatives (*FN*), for the number of incorrectly classified cleavage sites.

Sensitivity (*S_n_*) and Specificity (*S_p_*), which measures the capability of the model to correctly classify the cleavage sites and non-cleavage sites respectively, were computed as well:

To measure the overall model performance, we computed Accuracy (*A_cc_*):

In addition, we plotted the receiver operating characteristic curve (ROC) and computed the area under the curve (A_ROC_) for threshold independent evaluation. To compare against the prediction model developed by Barkan *et al*., we further determined the critical points on the ROCs of our SVM classifiers, which are defined as the points where the ROC curves intersect the lines connecting coordinates (1, 0) and (0, 1) on the graphs.

## Competing interests

The authors declare that they have no competing interests.

## Authors’ contributions

LJKW conceptualized the study and managed the technical aspects of the project. EPSE assisted with the preparation of data and analysis of results. LFPN and JCT contributed with ideas and assisted with the manuscript preparation. All authors read and approved the final manuscript.

## Supplementary Material

Additional File 1 **Dataset of granzyme B cleavage sites** This file contains the dataset of granzyme B cleavage sites. Training and test set sequences are listed on different tabs. Click here for file

Additional File 2 **SVM parameter optimization** Training sets of different sequence window datasets were trained under 10-fold cross-validation using various combinations of C and γ values. The optimal C and γ values for each training set are used to train the final SVM classifier. Optimal values are indicated below the corresponding chart. Click here for file
