# Supplementary material for: In silico prediction of the granzyme B degradome
Source: BMC Genomics. 2011 Nov 30;12(Suppl 3):S11. doi: 10.1186/1471-2164-12-S3-S11 (PMC3333170; doi:10.1186/1471-2164-12-S3-S11)
Supplement: Additional File 2 — SVM parameter optimization Training sets of different sequence window datasets were trained under 10-fold cross-validation using various combinations of C and γ values. The optimal C and γ values for each training set are used to train the final SVM classifier. Optimal values are indicated below the corresponding chart. [file 1471-2164-12-S3-S11-S2.pdf]

SVM parameter optimization: Training sets of different sequence window datasets were trained under 10-fold cross-validation using various combinations of C and γ values. The optimal C and γ values for each training set are used to train the final SVM classifier. Optimal values are indicated below the corresponding chart.

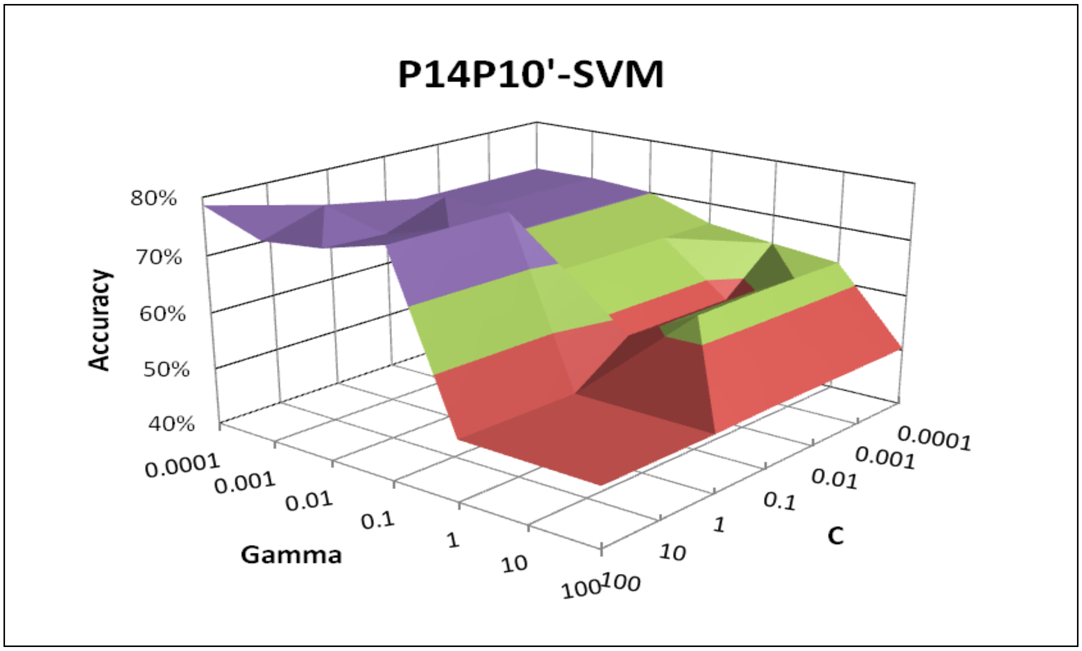

C=1 and γ =0.01

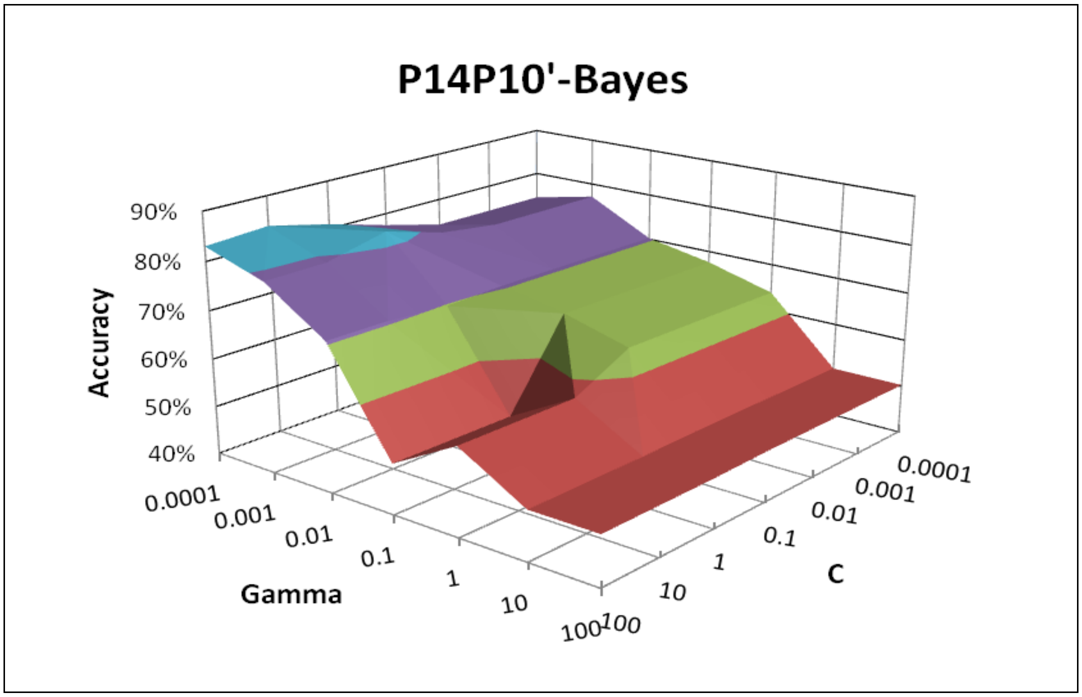

C=10 and γ =0.0001

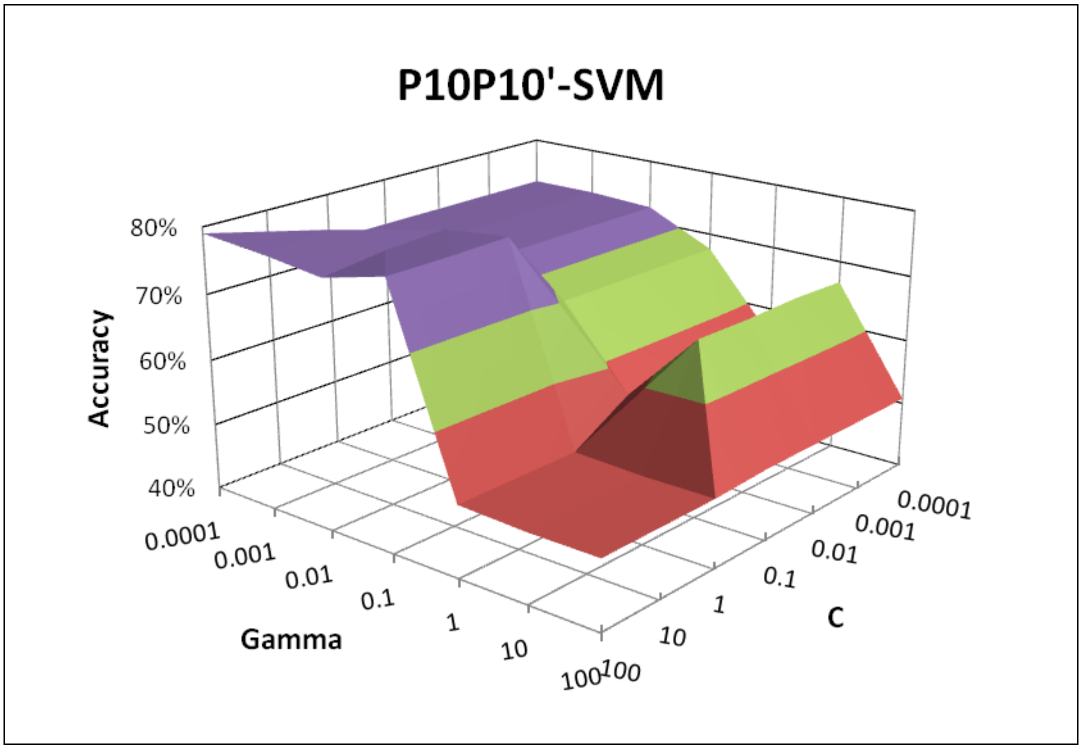

C=1 and γ =0.1

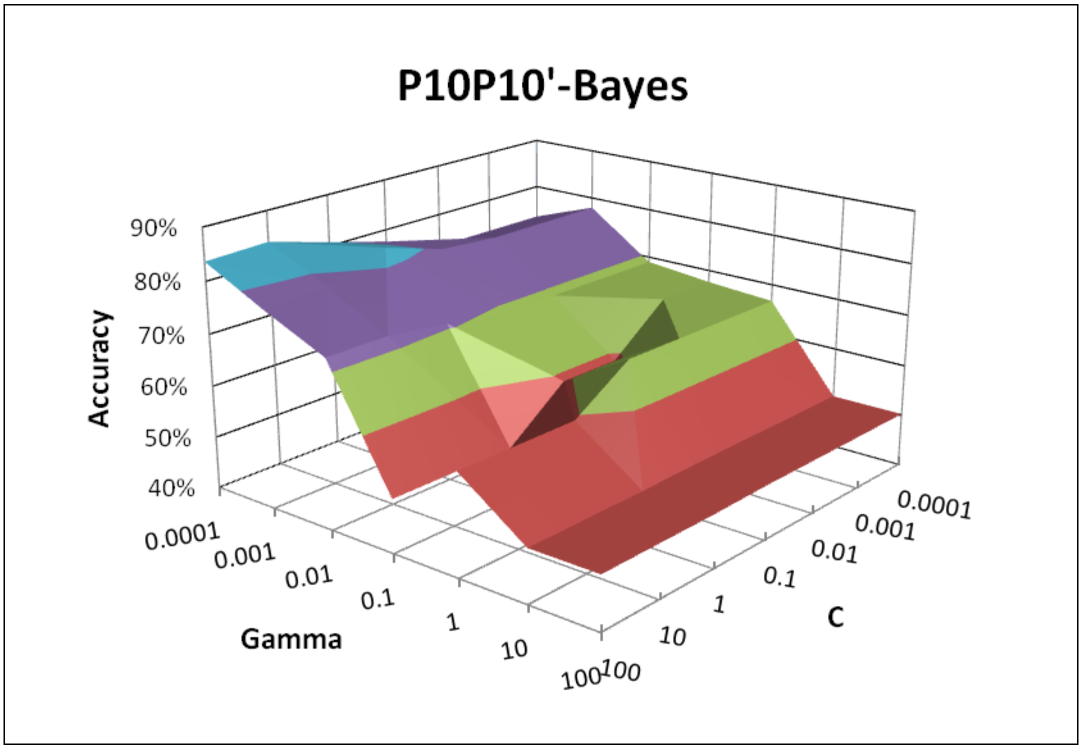

C=10 and γ =0.0001

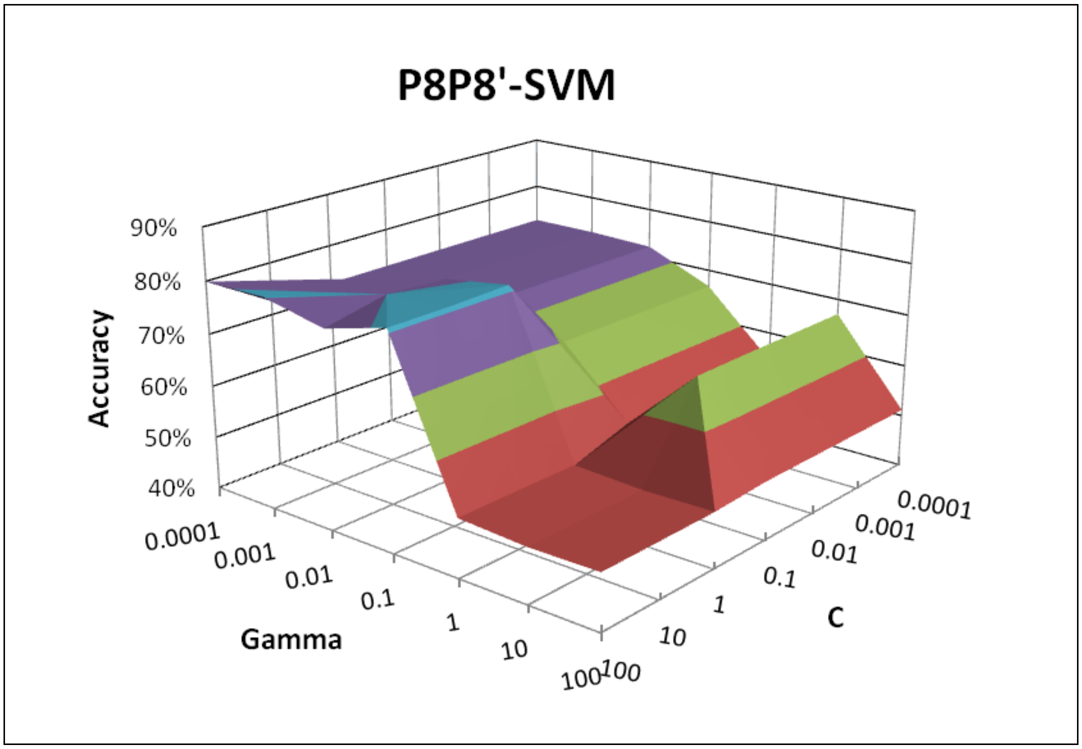

C=1 and γ =0.1

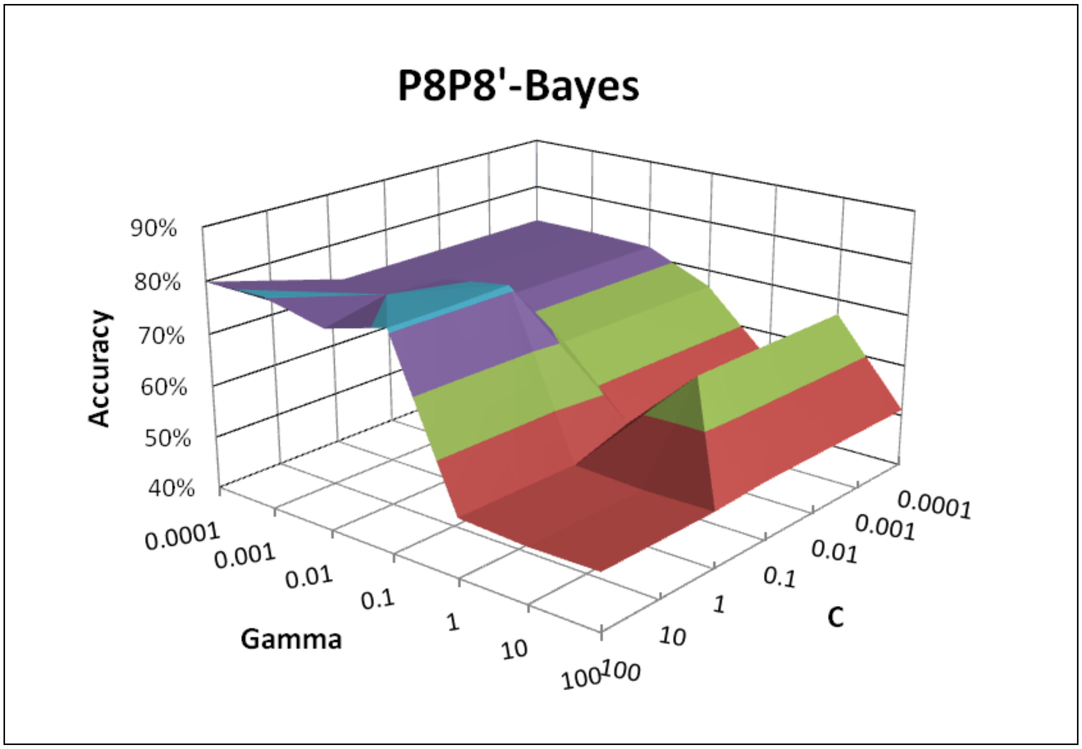

C=100 and γ =0.0001

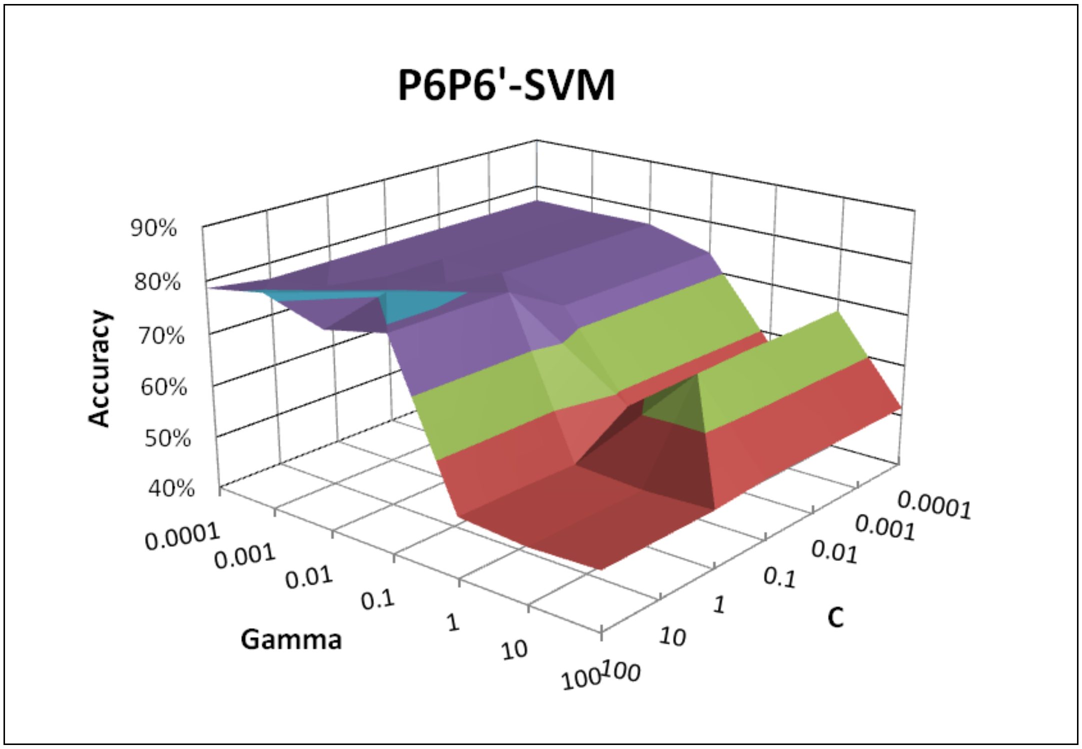

C=100 and γ =0.001

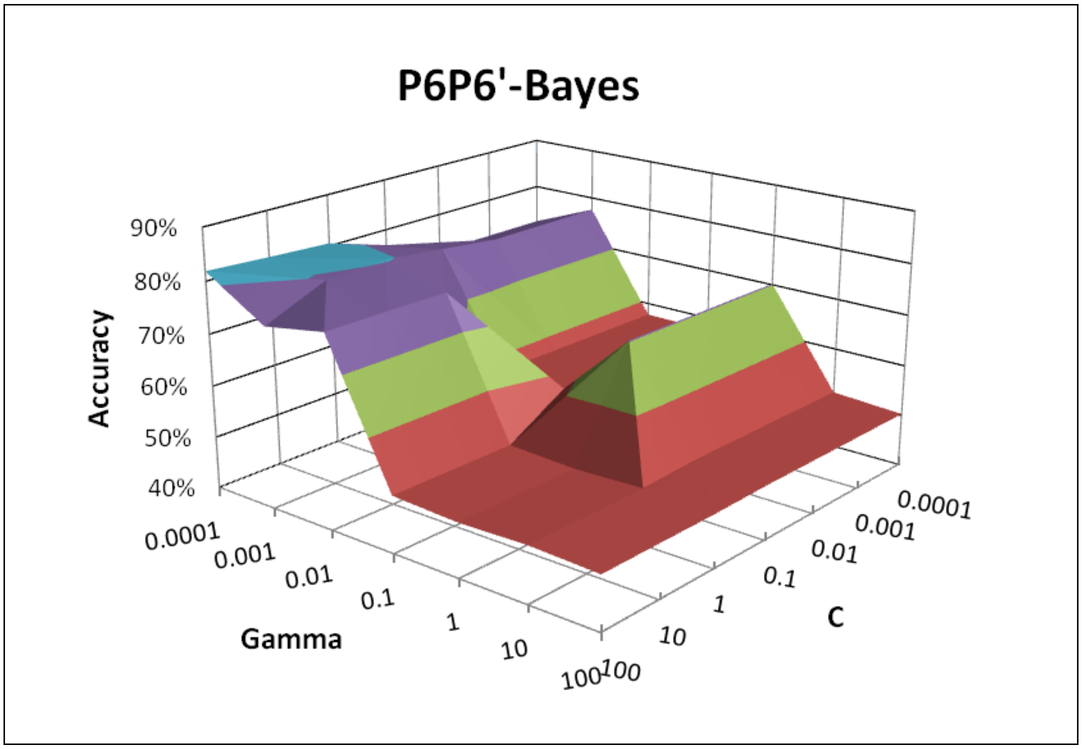

C=100 and γ =0.0001

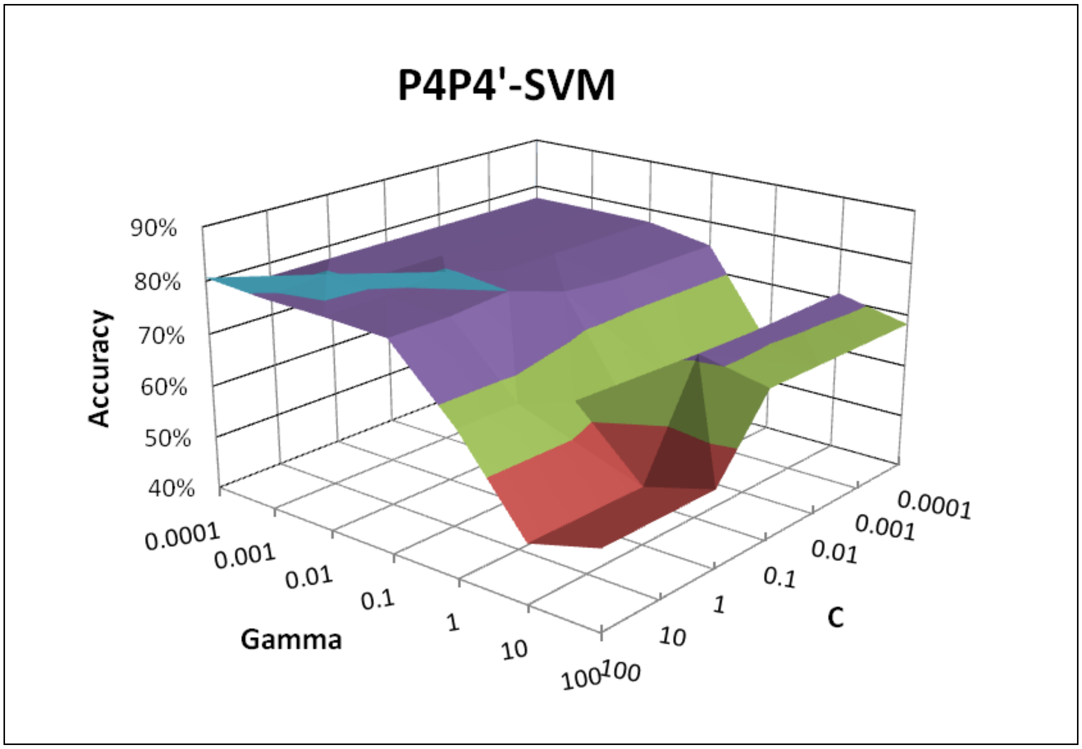

C=10 and γ =0.001

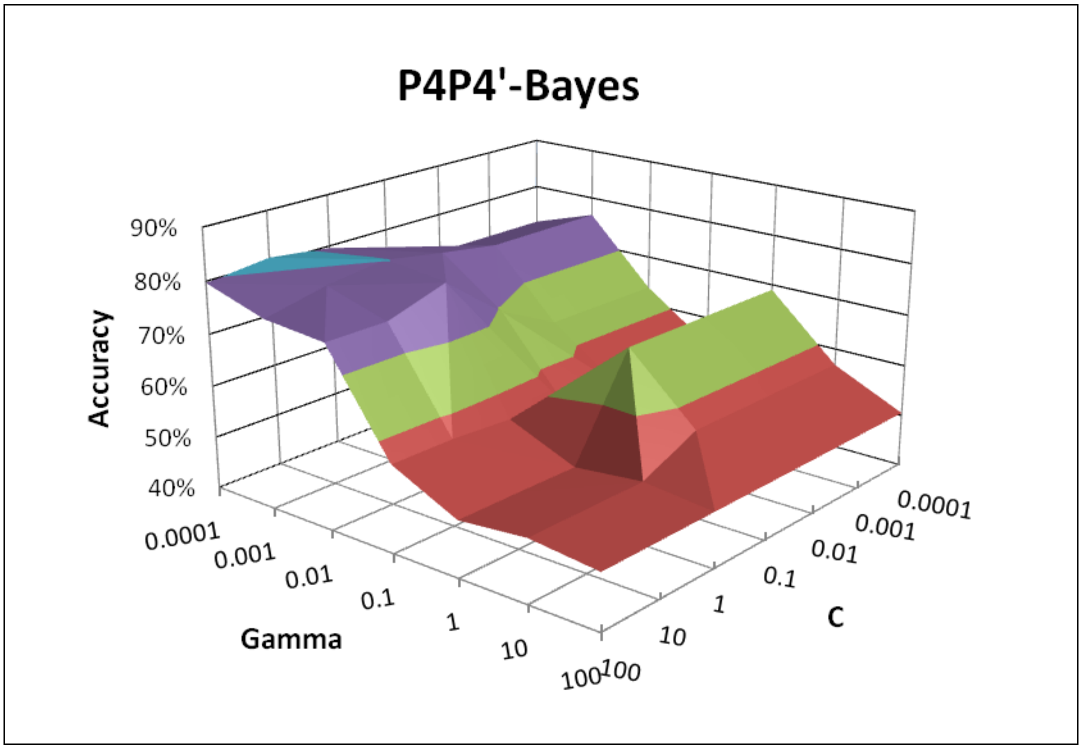

C=10 and γ =0.0001

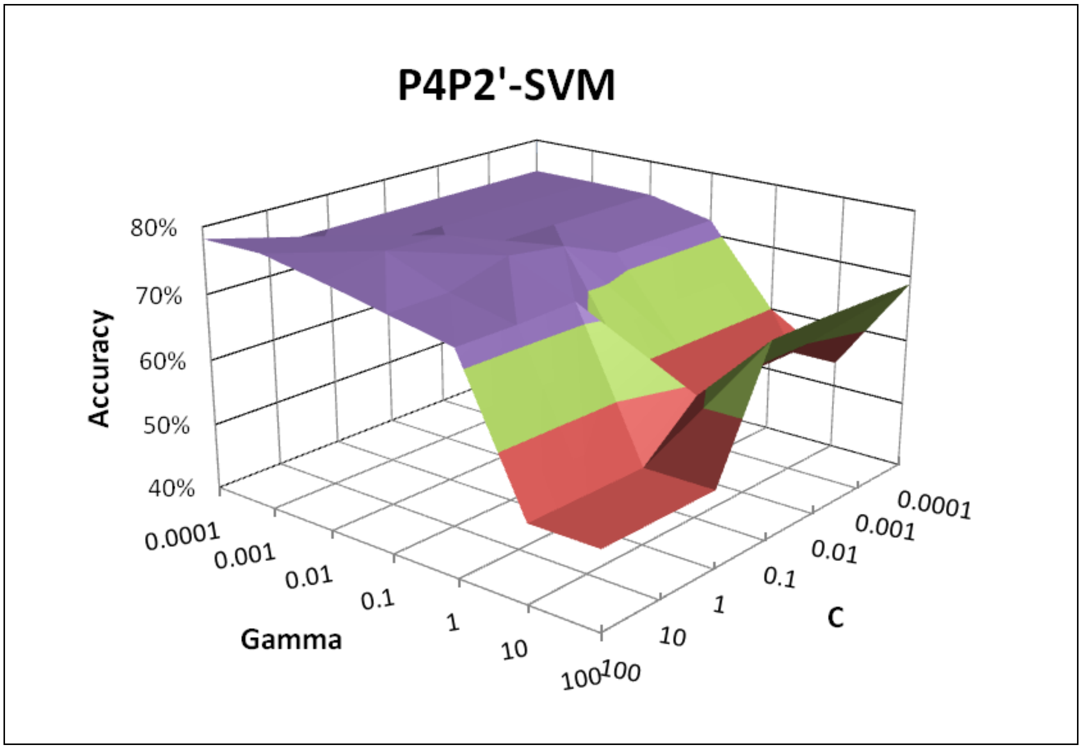

C=100 and γ =0.001

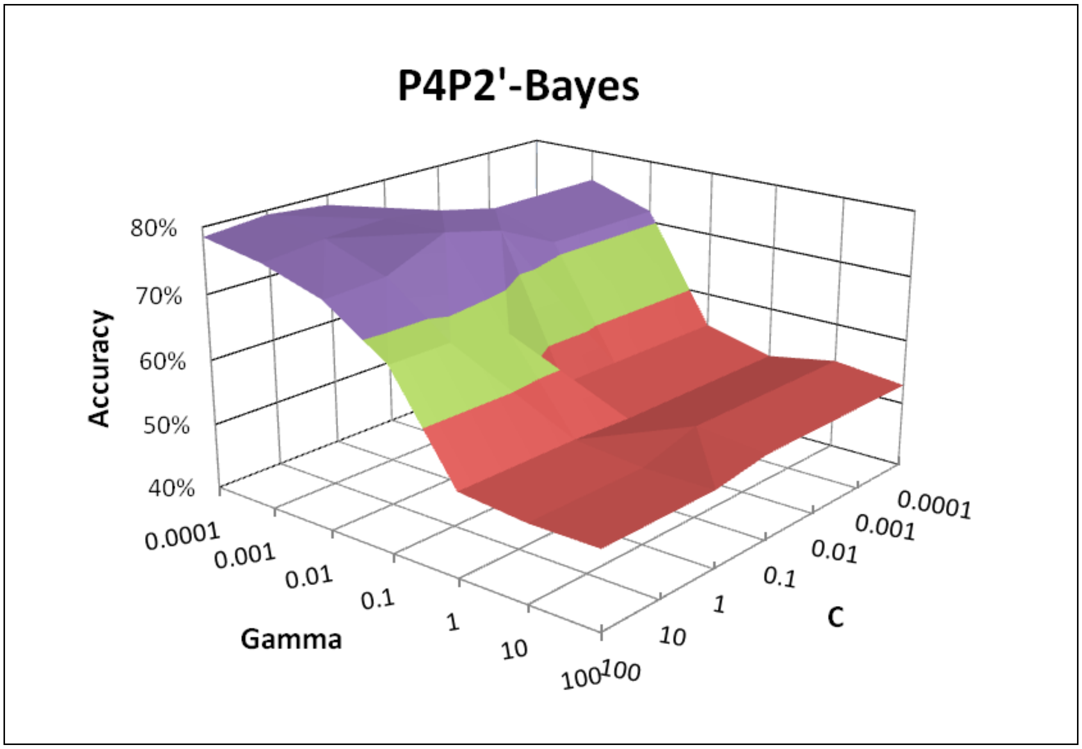

C=10 and γ =0.0001

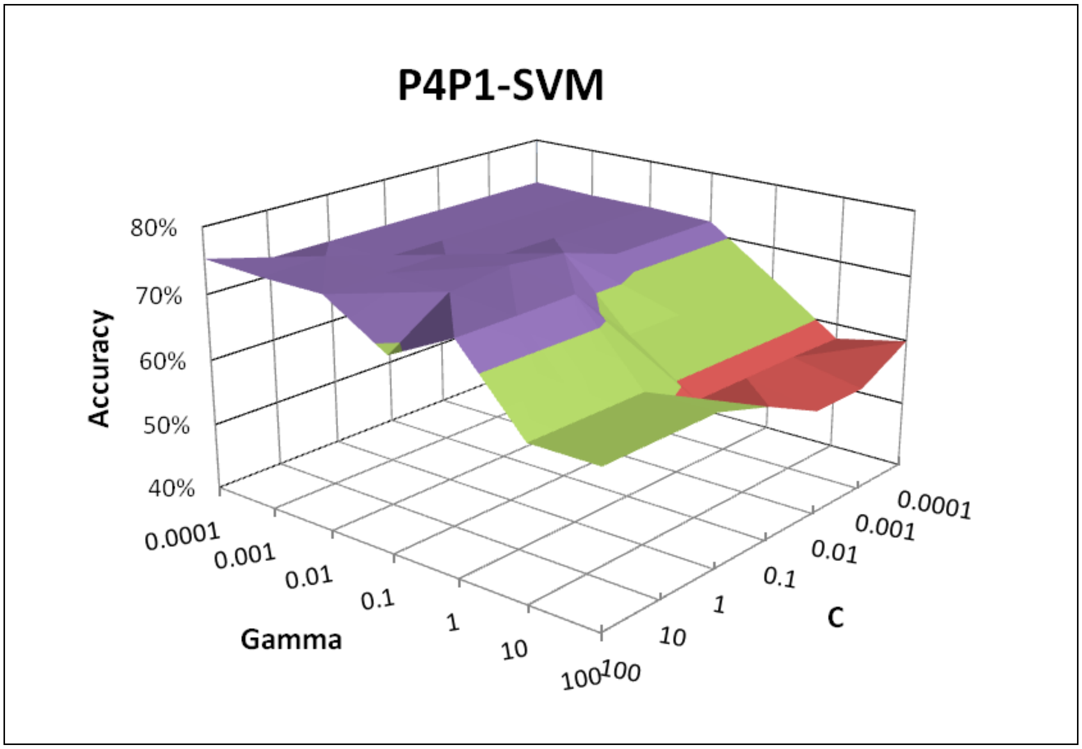

C=1 and γ =0.1

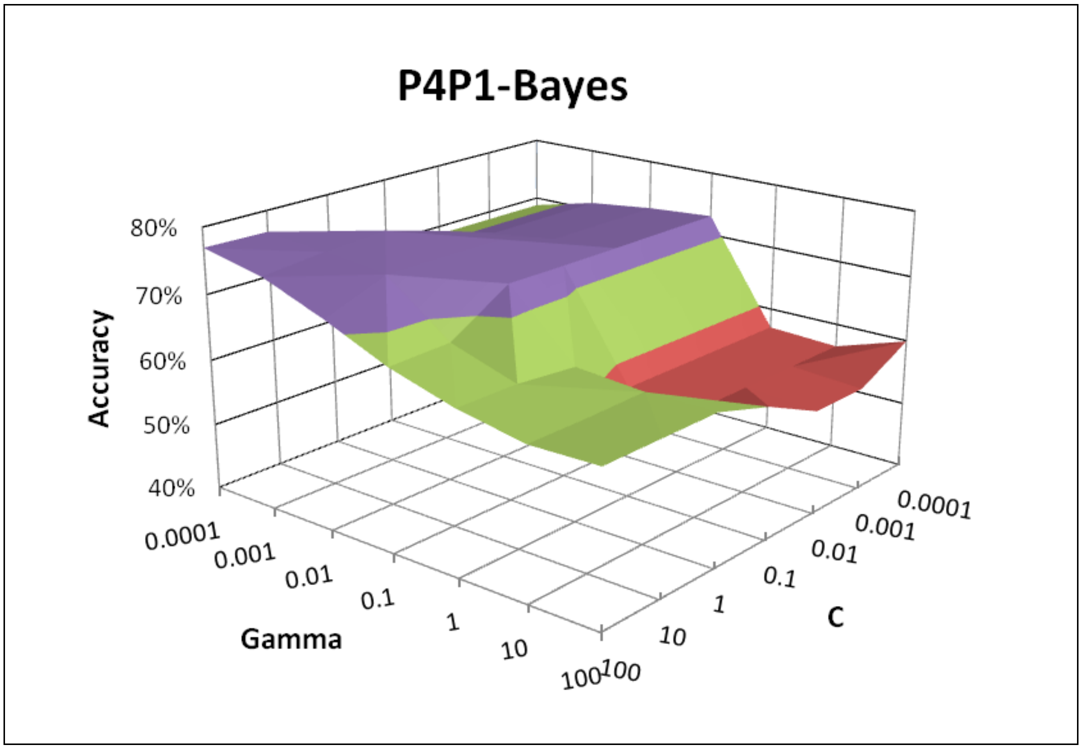

C=100 and γ =0.0001

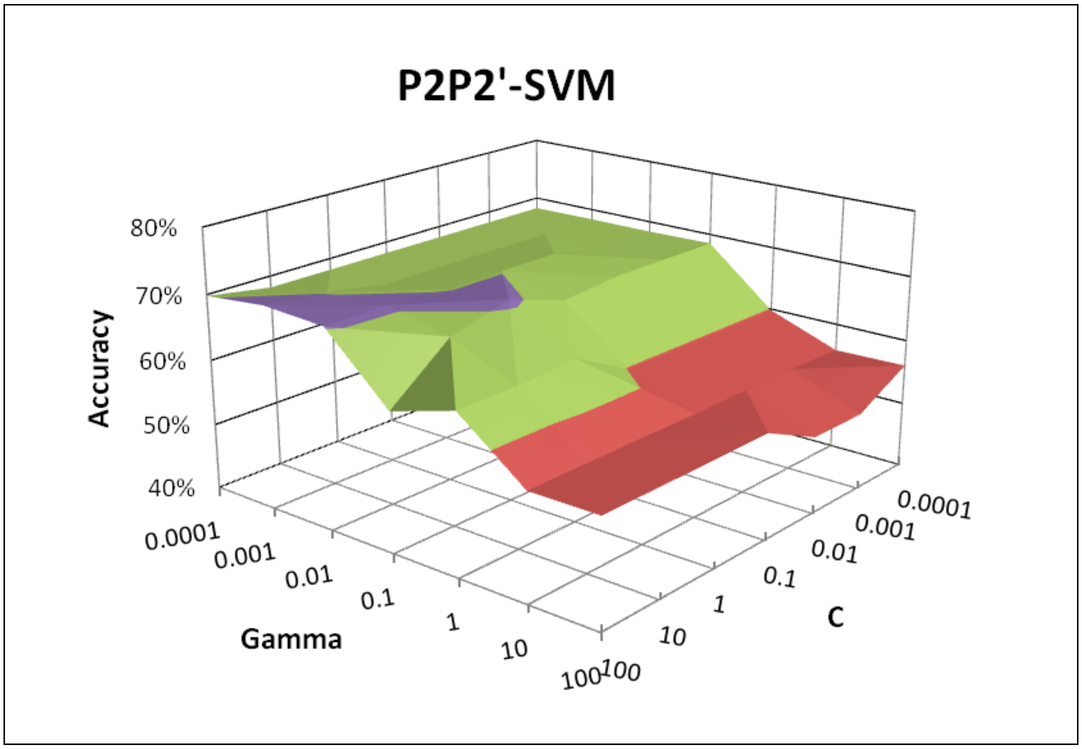

C=100 and γ =0.001

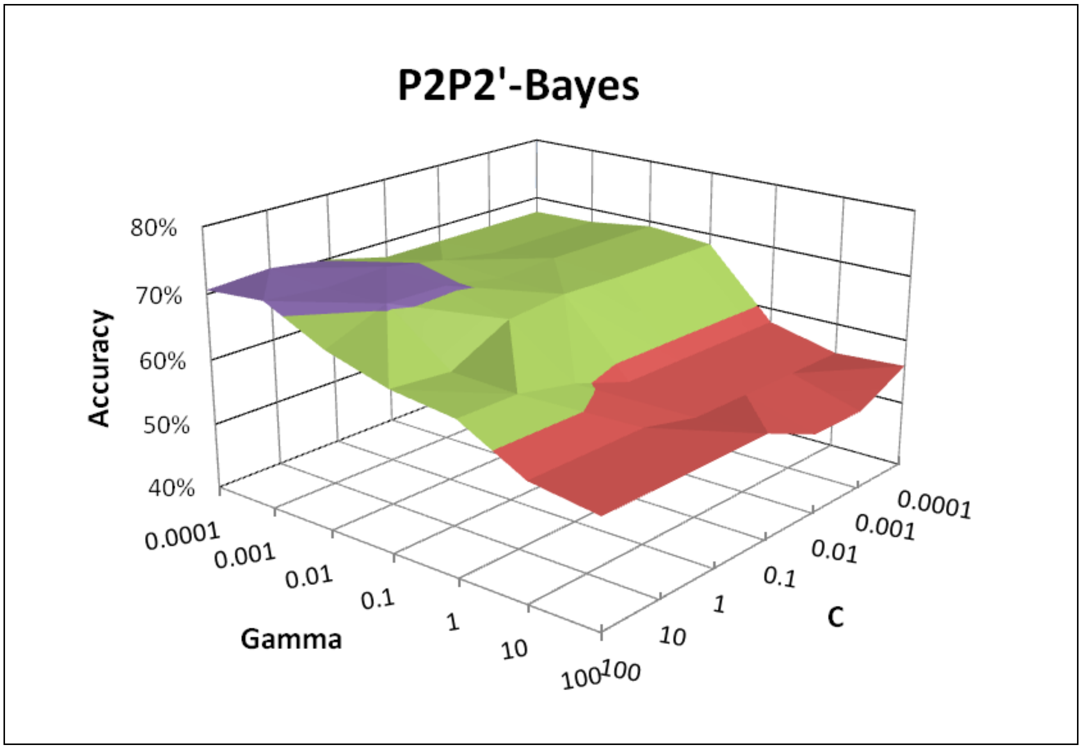

C=100 and γ =0.001
